# Insulin signalling regulates remating in female *Drosophila*

**DOI:** 10.1098/rspb.2010.1390

**Published:** 2010-08-25

**Authors:** Stuart Wigby, Cathy Slack, Sebastian Grönke, Pedro Martinez, Federico C. F. Calboli, Tracey Chapman, Linda Partridge

**Affiliations:** 1 Institute of Healthy Ageing and Department of Genetics, Evolution and Environment, University College London, London WC1E 6BT, UK; 2 Department of Zoology, Edward Grey Institute, Oxford University, Oxford OX1 3PS, UK; 3 Max Planck Institute for Biology of Ageing, Gleueler Straße 50a, 50931 Cologne, Germany; 4 Department of Epidemiology and Biostatistics, Imperial College, St Mary's Campus, London W2 1PG, UK; 5 School of Biological Sciences, University of East Anglia, Norwich, Norfolk NR4 7TJ, UK

**Keywords:** mating and reproduction, *Drosophila melanogaster*, sexual selection, fitness, nutrition, trade-off

## Abstract

Mating rate is a major determinant of female lifespan and fitness, and is predicted to optimize at an intermediate level, beyond which superfluous matings are costly. In female *Drosophila melanogaster*, nutrition is a key regulator of mating rate but the underlying mechanism is unknown. The evolutionarily conserved insulin/insulin-like growth factor-like signalling (IIS) pathway is responsive to nutrition, and regulates development, metabolism, stress resistance, fecundity and lifespan. Here we show that inhibition of IIS, by ablation of *Drosophila* insulin-like peptide (DILP)-producing median neurosecretory cells, knockout of *dilp2*, *dilp3* or *dilp5* genes, expression of a dominant-negative DILP-receptor (*InR*) transgene or knockout of *Lnk*, results in reduced female remating rates. IIS-mediated regulation of female remating can occur independent of virgin receptivity, developmental defects, reduced body size or fecundity, and the receipt of the female receptivity-inhibiting male sex peptide. Our results provide a likely mechanism by which females match remating rates to the perceived nutritional environment. The findings suggest that longevity-mediating genes could often have pleiotropic effects on remating rate. However, overexpression of the IIS-regulated transcription factor dFOXO in the fat body—which extends lifespan—does not affect remating rate. Thus, long life and reduced remating are not obligatorily coupled.

## 1. Introduction

Mating frequency has major fitness consequences for both sexes. For males, reproductive success typically increases linearly with the number of mates, but this may rarely be the case for females [1]. Although females can gain from mating with multiple males (e.g. [2–4]), matings can also incur costs (e.g. [5–8]), which may select for intermediate optimal mating rates [9]. For example, female insects often store insufficient sperm from a single mating to fertilize all the eggs produced over a lifetime [10], so remating is required to replenish sperm stores. However, because remating too frequently can result in reduced fitness [7,11,12], females should possess mechanisms to regulate remating rates optimally.

Nutrition is a key factor in the regulation of remating rates for female *Drosophila melanogaster*. Remating rates increase with the availability of high-quality nutrition (especially dietary yeast [13–15]) along with the rate of egg-laying and sperm use [16]. Nutrient-mediated receptivity status in females might therefore be a response to high egg-laying rates and to the depletion of sperm stores rather than as a direct response to nutrition. For example, post-mating increases in female feeding rates [17] occur as a response to egg-laying rather than as a response to the mating stimulus *per se* [18]. However, Harshman *et al*. [13] suggest that nutrition can influence mating behaviour directly (not just via egg production and sperm storage), an idea that is supported by the finding that a functioning ovary is not required for normal mating behaviour [18,19]. Thus, it is possible that mating rates are influenced more directly by nutrition than as a response to changes in egg-laying and sperm-use rates.

The insulin/insulin-like growth factor-like signalling (IIS) pathway is nutrient-responsive [20] and highly evolutionarily conserved [21]. IIS plays roles in development, metabolism, stress resistance, fecundity and lifespan across a broad range of taxa and is thus a key nutrient-sensing pathway [22,23]. Here we tested whether the IIS pathway plays a role in the regulation of female mating behaviour in female *D. melanogaster*. We measured the mating and remating rates of females in which the IIS pathway was genetically manipulated, focussing on IIS manipulations that result in extended or normal lifespan, to avoid mutations that might affect sexual behaviour via an overall reduction in female health. For a subset of mutants, we measured latency to mating in virgin matings, and fecundity in the 24 h following mating, to determine whether any changes in mating rates were coupled to changes in these traits. We also examined whether any effects of IIS on female remating were dependent on the receipt of the male ejaculate molecule, the sex peptide (SP)—the major male-derived effector of female sexual receptivity [24]. Sex peptide renders females unreceptive for up to several days following mating [25,26] by activating the female nervous system through the female SP receptor [27]. However, females can remain unreceptive for at least several hours in females mated to SP-lacking males ([25,26]; and see §3 below), suggesting that other seminal proteins can independently inhibit receptivity in the short term (e.g. DUP99B [28]; PEB II [29]). Our experiments determined whether SP was essential for IIS-mediated effects on female remating, or whether such effects could occur in the absence of SP.

## 2. Material and methods

### (a) Fly stocks

#### (i) Males

We used a laboratory-adapted, outbred, Q-type (contains inactive P-elements) fly strain, which was collected in Dahomey (now Benin) in 1970 and maintained since then in large population cages (e.g. [15]). Wild-type males were obtained from this stock. SP null males were *SP*^0^/*Δ*130 [26]. Both *SP*^0^ and *Δ*130 stocks were backcrossed into Dahomey [30].

#### (ii) Females

The genetic background for all experimental females was *white*^Dah^, which was derived by repeatedly backcrossing *w*^1118^ into Dahomey [31]. Mutations, inserts and *GAL4* drivers were backcrossed for five generations or more into *white*^Dah^.

#### (iii) IIS-mutant females

Three *Drosophila* insulin-like peptides (DILPs)—*dilp2*, *dilp3* and *dilp5*—are expressed in adult flies in the median neurosecretory cells (MNCs). Ablation of the MNCs was achieved in *dilp2GAL4/+; UAS*–*rpr*/+ flies, as described in [31]. Controls were *dilp2*GAL4/+ and UAS–*rpr/+*. Synaptic silencing of the MNCs was achieved by driving a *UAS-shi*^*ts*^ (temperature-sensitive) transgene [32] with *dilp2GAL4*. Experiments were conducted at the restrictive temperature (30°C) to silence MNCs in *dilp2GAL4/*+; *UAS-shi*^*ts*^/+ females. Controls were *dilp2GAL4/*+ and *UAS-shi*^*ts*^/+. To knockout *dilp2, dilp3, dilp5* and *dilp2–3* (double-knockout) genes, we used lines described in [33]. Two independent replicate lines of the *dilp2–3* double-knockouts (*dilp2–3^1^* and *dilp2–3^2^*) were used in two replicate experiments (see electronic supplementary material, table S1). Controls were *white*^Dah^ females.

Extracellular DILPs are transduced by the single insulin receptor (InR) to act on intracellular components of the IIS pathway. The activity of the InR was suppressed with a dominant-negative allele of *InR* (*InR*DN). Constitutive expression of *InR*DN was achieved in *UAS-InR*DN/+; *daGAL4/+* females as described in [34]. Controls were *UAS-InR*DN/+ and *daGAL4*/+. Adult-only expression of *UAS-InR*DN was achieved using an *actin* RU486-inducible P(Switch) GAL4 driver, *GS-255A* [35], to produce *InR*DN/+; *GS255A/+* females. Controls were *GS255A/*+. A loss-of-function mutant of an intracellular component of the IIS pathway, *Lnk*, which lies downstream of *InR*, is described by Slack *et al*. [36]. The transcription factor *dFOXO* is a negatively regulated, downstream target of the IIS pathway, which extends lifespan when overexpressed in the fat body [37,38]. Fat-body overexpression of *dFOXO* was achieved using the RU486-inducible P(Switch) GAL4 driver, S_1_106 [37]. Experimental females were *UAS-dFOXO*/+; S_1_106/+.

### (b) Fly culture

All flies were grown on standard sugar-yeast (SYA) food (e.g. [33,36]). Flies were maintained, and all experiments performed, at 25°C, except for the experiment using *UAS-shi*^*ts*^ flies, which was performed at the restrictive temperature (30°C). Adults were maintained on SYA food to which live-yeast granules were added (except for experiments involving RU486; see §2*c*). Males were grown at standard or at low larval density to minimize differences in adult body size, and were between 4 and 11 days post-eclosion at the time of experiments. For each experiment, the age differences between individual males were not more than 48 h. Males were separated from females at least 20 h before experiments and were randomly allocated to vials in pairs, which were then randomly allocated to treatments. Thus, differences in age or natural variation in body size among males were randomly distributed across treatments. Females were grown at standard density, collected as virgins within 8 h of eclosion, and aged for 4–6 days in groups of 10 prior to experiments.

### (c) RU486 experiments

When RU486 was delivered in the food in order to drive the expression of GAL4, the nutritional conditions used were as follows. In the first dFOXO experiment, females were maintained on SYA food containing 2× yeast concentration (with no live yeast added) after eclosion. Two days before the experiment half of the females were placed on 2× SYA food containing 200 µM RU486, to induce dFOXO overexpression. The remaining females were placed on identical food lacking RU486. In the second replicate dFOXO experiment, and in the InRDN experiment, 400 µM RU486 was delivered in the live yeast paste, as well as in 1× SY food. In these experiments, the females were placed on food with or without RU486 within 6 h of eclosion. Females were maintained on their respective food types up to and during the mating experiments.

### (d) Receptivity assay

Single females were placed with two wild-type males, or two *SP*^0^/*Δ*130 males for SP experiments, and allowed to mate once. After mating, males were discarded and females were maintained in single-sex groups of 10. The mated females were then placed individually with two wild-type males: for females mated initially to wild-type males this remating opportunity was at 24 h after their first mating, and for females initially mated to *SP*^0^/*Δ*130 males the remating opportunity was 5 h post-mating. The number of females that remated or did not remate within 1 or 2 h of exposure to males was recorded until at least 30 per cent of control females had remated. For several lines, replicate experiments were performed: raw data from replicate experiments are shown in the electronic supplementary material, table S1.

### (e) Latency to mating and fecundity assays

To measure latency to mating of virgin females, we recorded the time from when females were placed with two wild-type males until the time that mating started. Fecundity was measured over 24 h following mating. After mating, single females were placed in a fresh vial containing SYA food and live yeast paste. After 24 h females were removed from those vials and eggs were counted.

### (f) Statistical analysis

Data were analysed using R (v. 2.8.0) and jmp (v. 5; SAS Institute). Mating rate data (female remated versus female did not remate) were analysed with generalized linear models, all of which specified a binomial distribution for the mating data. When an analysis included replicated experiments and/or replicate mutant lines, we used generalized linear mixed effects models, specifying replicate experiment and/or line as random effects. For all models, to have direct and independent comparisons between treatments and controls, we specified the comparisons as linear orthogonal contrasts, which provided us with parameter estimates with corresponding *z*-values and two-tailed *p*-values (the results shown). The raw data for mating frequency are given in the electronic supplementary material, table S1. Fecundity data were analysed with one-way analysis of variances. Normality and detection of outliers were checked with Shapiro–Wilk and Grubbs tests, respectively. Latency to mating data, which could not be normalized by transformation, were analysed with Wilcoxon tests.

## 3. Results

### (a) Insulin signalling regulates female remating rate

**Figure 1.**
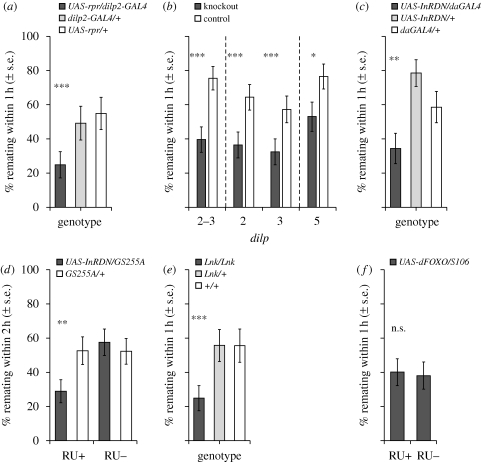
Remating frequency of IIS mutant and control females. The percentage of females remating within 1 or 2 h (±s.e.), 24 h after mating to wild-type males, is shown. Where experiments were replicated, or where independent replicate knockout lines were used (see electronic supplementary material, table S1), the mean value (±s.e) of replicate experiments/lines is shown. *p < 0.05 compared with controls; **p < 0.01; ***p < 0.001; n.s., non-significant. (*a*) MNC-ablated females (*UAS-rpr/dilp2-GAL4*, dark grey bar) and controls (*dilp2-GAL4/+*, light grey bar; *UAS-rpr/+*, white bar). (*b*) *dilp2–3, dilp2, dilp3* and *dilp5* knockouts (dark grey bars) and controls (white bars). Dashes separate different experiments. (*c*) Females constitutively expressing a dominant-negative *InR* (*UAS-InRDN/daGAL4*, dark grey bar) and controls (*UAS-InRDN/+*, light grey bar; *daGAL4/+*, white bar). (*d*) Females expressing the *UAS-InRDN* transgene at the onset of adulthood (by expression of the *GS255A* GeneSwitch driver induced with RU486) and controls (*UAS-InRDN/GS255A* females without RU486 (dark grey bars) and *GS255A/+* females with and without RU486 (white bars)). (*e*) *Lnk* mutant females (*Lnk^Del29^/Lnk^Del29^*, dark grey bar) and controls (*Lnk*^*Del29*^/+, light grey bar; +/+, white bar). (*f*) Females in which *dFOXO* was overexpressed in the adult fat body and controls. Expression was induced using RU486 to turn on a fat-body-specific GAL4 driver (*S_1_106*, dark grey bar). Control flies of the same genotype were maintained on RU486− food.

In their first (virgin) matings, virtually all females mated within 1 h, irrespective of genotype or treatment. However, we found striking differences in remating frequencies. Ablation of the MNCs significantly reduced female remating rate (*z* = 3.59, *p* = 0.0003; figure 1*a*). To investigate whether this effect was a result of loss of neuronal functions, we tested females in which the MNCs were synaptically silenced, using a dominant-negative *UAS-shi*^*ts*^ transgene: however, synaptic silencing of the MNCs did not significantly affect remating rate (*z* > 0.001, *p* > 0.99). Increased refractoriness in MNC-ablated females was therefore not due to the loss of the neuronal function of these cells, but more likely a result of reduced DILP levels. This hypothesis was supported by the finding that removal of any of the *dilp* genes expressed in the MNCs (*dilp2*, *dilp3* or *dilp5* genes [33]) had a similar effect on remating. Significantly fewer *dilp2–3* double-knockout females remated compared with controls (*z* = 8.08, *p* < 0.0001), as was the case for *dilp2* (*z* = 4.06, *p* < 0.0001), *dilp3* (*z* = 3.74, *p* = 0.0001) and *dilp5* (*z* = 2.01, *p* = 0.044) single gene-knockout females (figure 1*b*). Remating rates of *dilp2–3/+* heterozygote females were intermediate between controls and knockouts (*post hoc* comparisons: *dilp2–3* versus heterozygotes, *z* = 5.46, *p* < 0.0001; heterozygotes versus controls, *z* = 2.80, *p* = 0.005).

To test whether the effect on female remating rate is signalled through components of the IIS pathway downstream of the DILPs, we measured remating in females in which the activity of the DILP receptor (*Inr*) was suppressed. Ubiquitous constitutive expression of a dominant-negative *InR* transgene (*InRDN*), as well as ubiquitous post-developmental (adult only) expression [34], significantly reduced female remating compared with controls (constitutive, *z* = 2.98, *p* = 0.0028; adult-only, *z* = 2.62, *p* = 0.0087; figure 1*c*,*d*). This suggests that IIS-mediated changes in female remating rate are signalled via *InR*. An intracellular component of the IIS pathway, *Lnk* [36], which lies downstream of *InR*, is also involved in the regulation of female remating: *Lnk*-mutant females showed significantly reduced remating (*z* = 4.90, *p* < 0.0001; figure 1*e*). Finally, we examined remating rates in females that overexpress the insulin-responsive transcription factor, *dFOXO*, in the adult fat body. dFOXO transcriptional activity is downregulated in response to IIS and overexpression of *dFOXO* in the adult fat body is sufficient to extend lifespan [37,38]. However, we found no evidence for changes in remating rates in these females (*z* = 0.23, *p* = 0.81; figure 1*f*). Thus, overexpression of fat body *dFOXO*, though sufficient to extend lifespan, is insufficient to reduce remating.

### (b) The effect of insulin signalling on remating occurs independently of developmental defects, body size, fecundity and virgin latency to mating

Female mating behaviour could be affected by body size and/or fecundity (e.g. [39]). Thus, the reduction in body size and fecundity caused by developmental defects in some IIS mutants could potentially contribute to differences in remating [20,31,33,34,36]. However, restricting the expression of *InRDN* to adults also reduces female remating (figure 1*d*) in the absence of potentially confounding developmental defects, such as decreased body size [34]. Furthermore, *dilp3* and *dilp5* knockouts, which show reduced female remating (figure 1*b*), do not differ from controls in body size [33]. To evaluate the role of fecundity, we counted the eggs laid by *dilp2–3*, *dilp2* and *dilp3* knockouts in the 24 h between the first and second mating in the receptivity assays. Knockout of *dilp2–3* resulted in reduced fecundity (*F*_1,116_ = 29.7, *p* < 0.0001), but knockout of *dilp2* or *dilp3* did not affect fecundity (*F*_1,86_ = 0.39, *p* = 0.53 and *F*_1,84_ = 0.14, *p* = 0.71, respectively; electronic supplementary material, figure S1A). The fecundity of *dilp5* knockouts was not tested here, but lifetime egg production of *dilp5* knockout females was previously shown not to differ significantly from that of controls [33]. Together, these data indicate that IIS-mediated differences in remating can occur without corresponding differences in body size or fecundity.

Although we detected no effect of IIS on sexual receptivity in virgin females, our assay—counting the number of females mating within 1 h—was insensitive to potential differences in mating latency (the time from first exposure to males until the start of mating) occurring within that hour. To address this, we tested for effects of IIS on virgin latency to mating in *dilp2–3*, *dilp2* and *dilp3* knockout females. These lines were chosen because they show strong remating effects but small or no significant differences in body size and fecundity (see above; see also [33]). *dilp2–3* knockouts showed a small (approx. 30 s) but significant increase in mating latency relative to controls (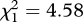, *p* = 0.004), but no significant differences were detected between either *dilp2* or *dilp3* single knockouts and controls (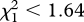, *p* > 0.2; electronic supplementary material, figure S1B). Thus, the removal of multiple *dilp* genes had a small effect on willingness of virgin to mate, but the removal of single *dilp* genes had none. We therefore conclude that IIS primarily affects female remating receptivity rather than latency to mating in virgin females, suggesting that IIS may interact with behavioural pathways that are initiated post-mating.

### (c) DILPs can influence remating rate independently of male SP

**Figure 2.**
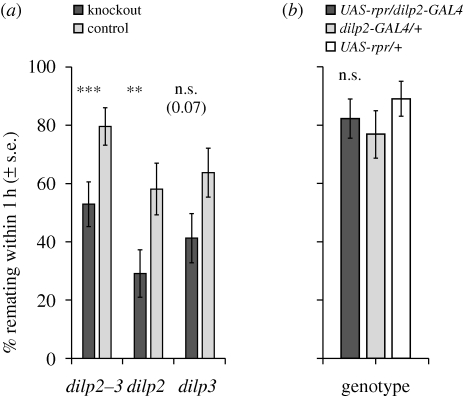
Female remating rate 5 h after mating to *SP*^0^ males. Where experiments were replicated (see electronic supplementary material, table S1) mean values of replicates (±s.e.) are shown. **p < 0.01 compared with controls; ***p < 0.001; n.s., non-significant. (*a*) *dilp2–3*, *dilp2* and *dilp3* females (dark grey bars), and controls (light grey bars). (*b*) Females with ablated MNCs (*UAS-rpr/dilp2-GAL4*, dark grey bar) and controls (*dilp2-GAL4*/+, light grey bar; *UAS-rpr*/+, white bar).

To investigate whether the effect of IIS on female mating behaviour is dependent upon the receipt of SP, we conducted receptivity tests following matings to males that produce no SP (*SP*^0^ males). We focused on the DILPs for these assays because (i) these molecules are upstream in the IIS pathway, and thus manipulations of the DILPs should also reduce downstream IIS; and (ii) *dilp* mutants show little or no differences from controls in body size and fecundity, and therefore their responses are unlikely to be confounded by differences in these traits (electronic supplementary material, figure S1A; [33]). We measured the remating rate of MNC-ablated females and controls 24 h after mating to *SP*^0^ males and found that all females—regardless of genotype—remated (*n* = 24–30), suggesting that SP is required for DILPs to affect remating over this timescale. Next, we examined remating rates in the absence of SP over a shorter timescale, 5 h after initial matings, at which time a proportion of control females show non-receptivity, presumably as an effect of non-SP receptivity-inhibiting ejaculate molecules [28,29]. Remating rates were significantly lower for *dilp2–3* (*z* = 3.54, *p* = 0.0004) and *dilp2* knockouts (*dilp2*, *z* = 2.29, *p* = 0.022), and marginally non-significantly lower for *dilp3* knockouts (*z* = 1.84, *p* = 0.066; figure 2*a*), showing that the DILPs can affect female remating behaviour in the absence of SP over short timescales. Unexpectedly, however, MNC-ablated females (which have reduced levels of DILPs 2, 3 and 5) did not show reduced remating rate in the absence of SP (*z* = 0.57, *p* = 0.57; figure 2*b*). Thus, while the absence of DILPs 2 and 3 inhibits female remating in the absence of SP, reducing the levels of these same DILPs by ablation of the MNCs has no detectable effect. One possible explanation for this result is that the low quantities of DILPs that are produced in MNC-ablated females [31] are sufficient to allow normal remating in the absence of SP (i.e. the effect of overall DILP dose on female remating rate requires SP). Another possibility is that other functions of the MNCs might play a role in the female response to receptivity-inhibiting components of the seminal fluid other than SP. Thus, the removal of a proportion of the MNCs might disrupt the normal behavioural responses to lowered DILP levels. Our results are consistent with the general finding that ablation of the MNCs produces effects beyond those of DILP 2, 3 and 5 removal. For example, MNC-ablated females display elevated starvation resistance [31], whereas *dilp2–3,5* knockouts are not resistant to starvation [33].

## 4. Discussion

### (a) IIS regulation of remating

Our results show that key components of the IIS pathway regulate female remating rate in *D. melanogaster*, suggesting that a major mechanism by which females adjust their mating behaviour in response to nutrition is via IIS. Thus, we provide a likely molecular basis for the link between nutrition and sexual behaviour in *Drosophila* [13–15]. Furthermore, the effects of IIS on female remating can—at least to some extent—act independently of SP, the major male-derived molecular effector of female receptivity. This finding is consistent with the lack of interaction effects between nutrition and SP on female mating rate found by Fricke *et al*. [30]. These two major regulators of female remating, IIS and SP, are likely to signal the normal requirement for remating in response to factors that limit female reproduction, namely nutrients required to produce eggs [40] and sperm required for fertilization [41]. This dual mechanism for controlling remating, via IIS and SP, may enable female mating rate to most effectively match reproductive opportunities while avoiding costly superfluous matings [6,11,42].

Females may benefit unconditionally from their first mating as they need to obtain sperm to fertilize eggs. Thus, the lack of effect of IIS on virgin receptivity may be because sexually mature females gain from a rapid first mating—and there is no benefit to delaying mating—whatever may be the nutritional conditions. However, in *D. melanogaster*, as in many insects, a single mating fails to provide sufficient sperm to fertilize all the eggs produced over a lifetime [16], meaning that females must remate to replenish sperm stores (e.g. [41]). A tighter calibration of nutrition with remating rate may be beneficial following the first mating, because nutrition affects female fecundity and the rate of sperm use [16] such that, under poor nutritional conditions, females will need to replenish stored sperm (i.e. mate) less frequently [13]. Hence, the regulation of female remating receptivity in response to nutritional status is likely to be key for female fitness [14,15].

The sexual behaviour of IIS mutant females broadly mimics that of females on a poor diet [13–15], which is consistent with the hypothesis that reduced IIS partly (though not wholly) mimics dietary restriction. Like reduced IIS, restriction of dietary nutrients can result in increased lifespan (reviewed in [43]) and decreased mating rates [13–15]. Manipulating components of the IIS pathway, as performed here, could generate a mismatch between the perceived and real nutritional environment, resulting in potentially sub-optimal mating rates for a given rate of egg-laying. However, it is clear that there is no obligatory link between egg-laying and mating rate, because females that lack the ability to produce eggs display normal mating and remating behaviours [18,19,44]. Moreover, our study shows that females can possess normal fecundity but show reduced mating rates under IIS suppression (figure 1*b*; electronic supplementary material, figure S1A).

### (b) Insulin signalling, mating rate and lifespan

Lifespan can be extended by genetic manipulations that reduce IIS, including several mutants used in this study (MNC-ablated [31]; *dilp2* and *dilp2–3* [33]; *InRDN* [34]; *Lnk* [36]). However, lifespan can also be extended by reducing mating frequency [4,6,18]. Our results therefore highlight the importance of controlling mating rates in studies that investigate the genetics of ageing, to avoid confounding effects of differential sexual activity on lifespan. Our discovery that several IIS manipulations that increase lifespan also increase the inter-mating interval raises an important potential confound regarding the conclusions of ageing studies in which flies are maintained in mixed sex groups (e.g. [45–47]). Reduced mating rates in experimental mutant lines could potentially confound ageing studies because females might live longer owing to reduced mating rates rather than as a direct effect of the genetic manipulations themselves. The solution to this potential confound is to control mating rates in lifespan studies in order to test for direct effects on lifespan [48]. However, the results from our dFOXO experiment (figure 1*f*) show that it is also possible to uncouple the regulation of female sexual behaviour and the regulation of lifespan, in accordance with the uncoupling of lifespan and fecundity [38,49]. Thus, both behavioural and physiological aspects of reproduction can be uncoupled from lifespan extension under certain conditions.

### (c) Pathways through which insulin signalling regulates remating

The effects of single *dilp* mutants on remating were, surprisingly, only marginally weaker than the effects of MNC ablation or *dilp2–3* double mutants, despite the apparently weaker genetic intervention (figures 1 and 2). However, ablation of the MNCs is incomplete, and DILP levels are reduced rather than abolished in the flies we used [31]. Moreover, there is compensation and synergism between DILPs such that knockouts of single *dilp* genes can affect the expression of one or more of the other *dilps* [33]. For example, *dilp2* and *dilp2–3* mutant flies exhibit increased expression of *dilp5*, while *dilp3* mutants exhibit reduced levels of *dilp2* and *dilp5* expression [33]. Such effects could explain the relatively strong phenotypes of the single *dilp* knockouts in comparison with the *dilp2–3* knockout and MNC-ablated females.

The extracellular DILPs, the InR and the intracellular IIS component, Lnk, all regulate female remating rate, but it is currently unclear which downstream molecules are involved. A major downstream target of the IIS pathway is the transcription factor dFOXO, but we found no effect of fat body dFOXO expression on female mating. One possibility is that dFOXO mediates the effect of reduced IIS on remating rates in tissues other than the fat body. Another possibility is that the effect of IIS on remating rate occurs via the target of rapamycin (TOR) pathway. The TOR pathway senses amino acids and runs parallel to, and interacts with, IIS [23]. The IIS and TOR pathways interact to control growth, and TOR signalling, like IIS, has been shown to regulate lifespan [50]. Moreover, recent work shows that the TOR pathway is involved in mating-induced changes in diet choice [51,52], supporting the idea that TOR functions in the coordination of behavioural responses to mating and the nutritional environment. It will be important to investigate the mating behaviour of TOR-pathway mutants to determine whether this pathway is involved in the regulation of mating and whether the effects of IIS on female remating are mediated through TOR signalling. It will also be important to determine through which tissues IIS regulates remating.

## 5. Conclusions

Our work shows that components of the IIS pathway modulate sexual behaviour by significantly altering the receptivity of mated female *D. melanogaster*. Thus, we provide a likely molecular basis for the link between nutrition and sexual behaviour in insects, which is an important step in understanding the mechanisms underlying life-history traits and trade-offs. Reproduction and nutrition are linked across a broad range of taxa, including mammals [53,54], and many of the effects of IIS (e.g. on lifespan and fecundity) are highly evolutionarily conserved. We conclude that the regulation of mating behaviour via IIS could be common among animals.
